# A rare case of isolated unilateral pulmonary vein atresia presenting as interstitial lung disease in a young adult

**DOI:** 10.34172/jcvtr.025.33139

**Published:** 2025-09-28

**Authors:** Arnav Shandil, Mansi Verma, Sushma Makhaik, Sumala Kapila

**Affiliations:** Department of Radiodiagnosis, Indira Gandhi Medical College, Shimla-171001, India

**Keywords:** Pulmonary vein, Interstitial lung disease, Adult

## Abstract

Unilateral pulmonary vein atresia is an unusual congenital cardiovascular abnormality. It occurs due to failure of incorporation of pulmonary veins into the left atrium. It is usually diagnosed in childhood and diagnosis after adulthood is very rare. Herein we present a case of 21-year-old young adult with isolated unilateral pulmonary vein atresia who presented with unilateral interstitial lung disease.

## Introduction

 Unilateral pulmonary vein atresia is a rare congenital anomaly which generally presents in infancy or childhood with pneumonia or recurrent hemoptysis.^1^ Cases of adult presentation are reported but are uncommon. The proposed mechanisms include presence of diaphragm completely occluding the veno-atrial junction or atresia of the extra pulmonary venous segment.^2^ It can be isolated or associated with other cardiovascular anomalies in 50% of the cases. ^3^

## Case Presentation

 A 21-year-old male presented with occasional dyspnea on exertion for 3 months. The patient did not have any respiratory symptoms during childhood with non-significant past medical history. There was no previous history of admission. He was afebrile and acyanotic with arterial oxygen saturation of 93%. Echocardiography revealed small left pulmonary artery with normal cardiac chambers, no valvular regurgitation and normal gradients across pulmonary valve.

**Figure 1:**
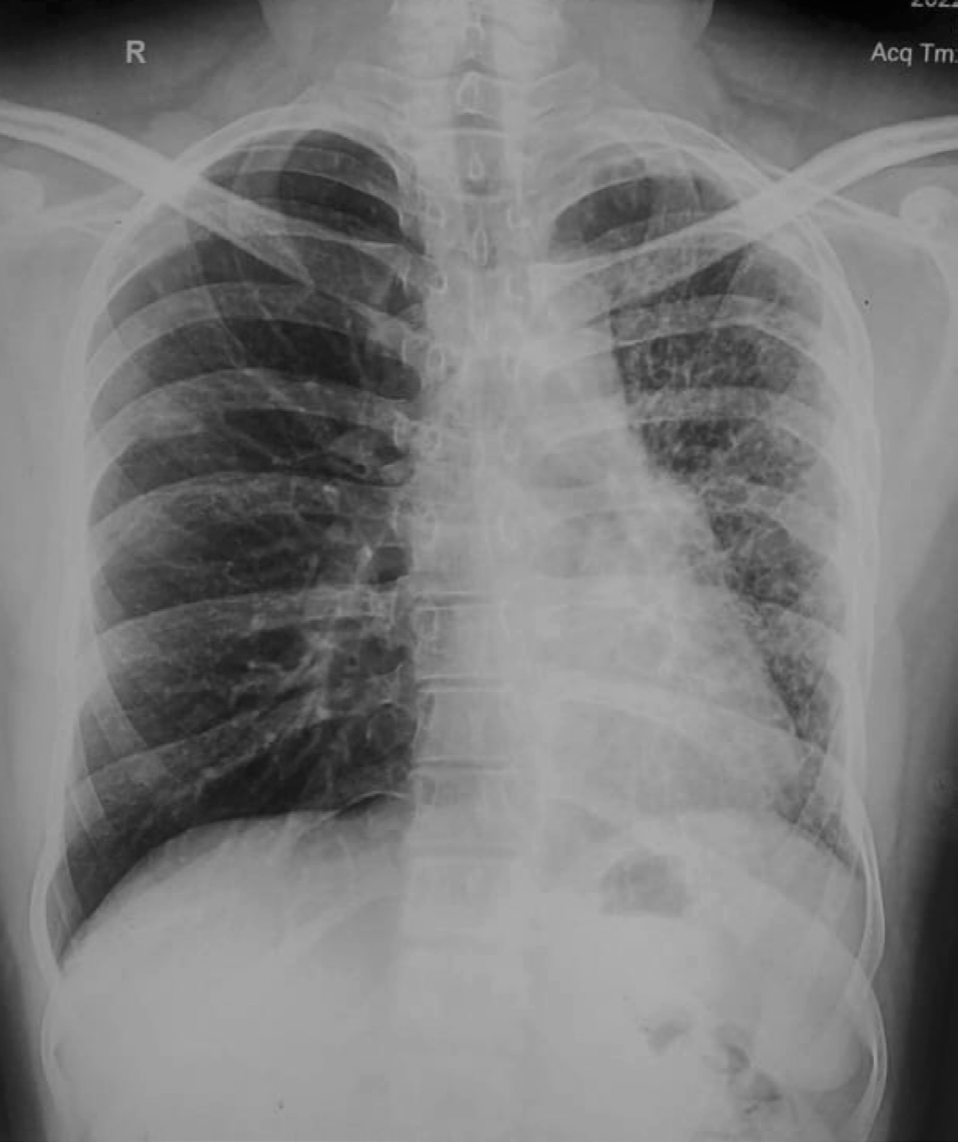
Chest X-ray shows reduced volume of left hemithorax with ipsilateral mediastinal shift. Diffuse reticular opacities are noted in left lung

**Figure 2:**
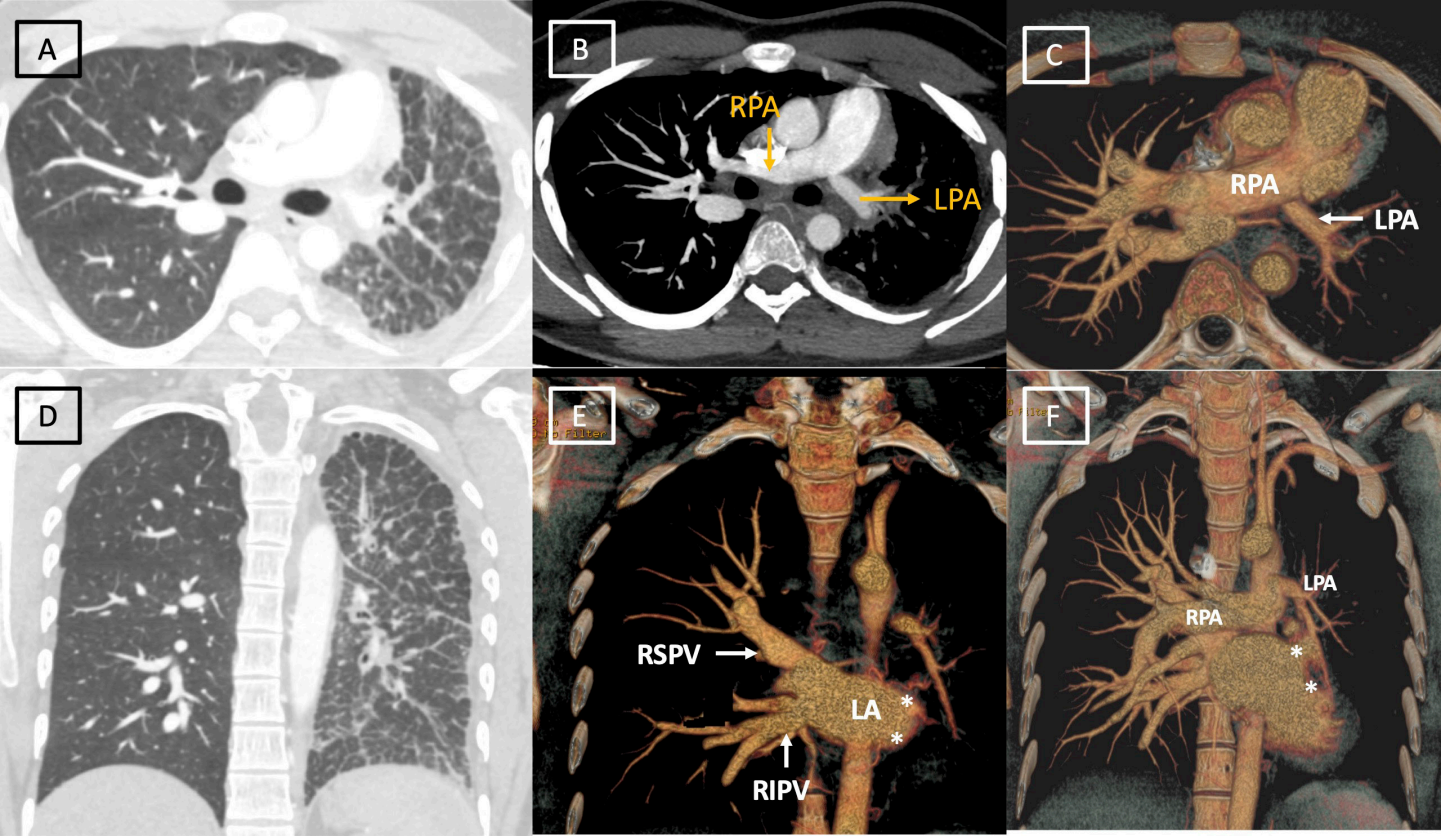
Axial (A) and coronal (D) lung window images show diffuse interlobular septal thickening, areas of ground glass attenuation and peri bronchovascular thickening. On CT angiography axial (B) and volume rendered images (C) there is small left pulmonary artery and normal main and right pulmonary artery. Coronal volume rendered (E and F) images shows absent pulmonary veins on the left side and a smooth lateral aspect of the left atrium with hypoplastic ostia of left superior and inferior pulmonary veins (* in D and E). Normal right pulmonary venous drainage is seen on the right side. RPA: Right pulmonary artery; LPA: Left Pulmonary Artery; RSPV: Right Superior Pulmonary Vein; RIPV: Right Inferior Pulmonary Vein; LA: Left Atrium

 Chest radiograph (Figure 1) demonstrated decreased volume of left hemithorax with diffuse reticular opacities and slight ipsilateral mediastinal shift. Computed tomography done for further evaluation revealed decreased left lung volume, ipsilateral mediastinal shift with interlobular septal thickening and areas of ground glass attenuation. **(**Figure 2 A, D) Interestingly, the left pulmonary artery was hypoplastic. (Figure 2 B, C) The right pulmonary artery was relatively dilated. However, there was absence of abrupt narrowing of peripheral pulmonary vessels, right ventricular hypertrophy or enlargement, thus excluding pulmonary artery hypertension.Pulmonary venous drainage to left atrium was normal on right side, however pulmonary veins were not visualized on left side with smooth lateral border of left atrium. (Figure 2 E, F) No bronchial obstruction was seen. No associated cardiac or venous anomaly was seen. Right heart catheterization revealed normal right pulmonary artery and right sided pressures. As the patient did not have any significant respiratory, cardiac or systemic symptoms, he is currently managed conservatively and is on follow up. Further plan is to perform left pneumonectomy as it will alleviate the nidus of infection and will remove the dead space causing exercise intolerance.

## Discussion

 Isolated unilateral pulmonary venous atresia (UPVA) is a rare congenital abnormality. To the best of our knowledge, few adult cases of isolated UPVA have been reported. UPVA results when common pulmonary vein fails to incorporate into the left atrium. This leads to inadequate gas exchange at the alveolar level leading to poor ventilation and oxygenation of the affected lung. Owing to markedly reduced flow, the ipsilateral pulmonary artery in the affected lung is underdeveloped with flow reversal towards the contralateral side and development of systemic to pulmonary artery collaterals.^4^

 UPVA is associated with congenital heart defects like ventricular septal defect or double superior vena cava in approximately 50% of patients.^4^ On histological examination, these veins show intimal fibrosis without any inflammatory reaction.^5^ The most common presenting symptoms include recurrent infections of the respiratory tract and hemoptysis due to the systemic collateral supply to the affected lung. ^6^ The imaging findings on computed tomography include small hemithorax, a diminutive ipsilateral pulmonary artery and absence of ipsilateral pulmonary vein drainage into the left atrium. Parenchymal findings include ground-glass opacities and septal thickening, likely representing dilated lymphatics and bronchial veins.^5^ Later, unilateral interstitial lung disease develops due to chronic pulmonary edema.^7^

 Unilateral pulmonary vein atresia is a diagnostic predicament, especially in adult patients. In presence of a confluent mediastinal soft tissue, lung malignancy and fibrosing mediastinitis that involves the hilar vessels, are the main differential diagnosis. However, the presence of a small hemithorax without any bronchial obstruction helps in diagnosing a congenital pathology. Pneumonectomy is the treatment option for alleviating the nidus of recurrent pneumonias and relieving significant left-to-right shunt.^5^ In long standing cases with severe pulmonary hypertension, lung transplantation is preferred.

## Conclusion

 UPVA is an unusual congenital anomaly with a diagnostic dilemma. Computed tomography aids in providing a definite diagnosis. Owing to variable clinical severity, the management has to be individualized in patients with no or mild symptoms.

## Competing Interests

 The authors declare no potential conflicts of interest with respect to research, authorship, and/or publication of this article.

## Ethical Approval

 As per institutional policy, ethical approval is not required for case reports.
